# Recent progress in Surface-Enhanced Raman Spectroscopy detection of biomarkers in liquid biopsy for breast cancer

**DOI:** 10.3389/fonc.2024.1400498

**Published:** 2024-07-08

**Authors:** Xiaobei Liu, Yining Jia, Chao Zheng

**Affiliations:** ^1^ Cheeloo College of Medicine, Shandong University, Jinan, China; ^2^ Department of Breast Surgery, The Second Hospital of Shandong University, Jinan, China; ^3^ Institute of Translational Medicine of Breast Disease Prevention and Treatment, Shandong University, Jinan, China

**Keywords:** SERS, biomarker, liquid biopsy, breast cancer, early diagnosis

## Abstract

Breast cancer is the most commonly diagnosed cancer in women globally and a leading cause of cancer-related mortality. However, current detection methods, such as X-rays, ultrasound, CT scans, MRI, and mammography, have their limitations. Recently, with the advancements in precision medicine and technologies like artificial intelligence, liquid biopsy, specifically utilizing Surface-Enhanced Raman Spectroscopy (SERS), has emerged as a promising approach to detect breast cancer. Liquid biopsy, as a minimally invasive technique, can provide a temporal reflection of breast cancer occurrence and progression, along with a spatial representation of overall tumor information. SERS has been extensively employed for biomarker detection, owing to its numerous advantages such as high sensitivity, minimal sample requirements, strong multi-detection ability, and controllable background interference. This paper presents a comprehensive review of the latest research on the application of SERS in the detection of breast cancer biomarkers, including exosomes, circulating tumor cells (CTCs), miRNA, proteins and others. The aim of this review is to provide valuable insights into the potential of SERS technology for early breast cancer diagnosis.

## 1 Introduction

In 2022, There will be an estimated 357200 new cases of breast cancer in China, resulting in 75000 deaths (1). Breast cancer is classified into four major molecular subtypes based on immunohistochemical classification: luminal A and luminal B (expressing the estrogen receptor (ER)), basal-like and human epidermal growth factor receptor 2 (HER2)-enriched (without ER expression) (2, 3). The choice of clinical treatment and prognosis is closely related to breast cancer subtypes, highlighting the importance of early detection. Patients with early-stage breast cancer have a survival rate of 98%, while late-stage patients’ survival drops to 27% (4). Triple-negative breast cancer is highly invasive and metastatic, associated with a poor prognosis and high recurrence rates (5), whereas the Luminal A/B subtype is suitable for hormone therapy because of endoplasmic reticulum enrichment (6). Breast cancer cells exhibit heterogeneity in molecular and morphological characteristics. However, conventional imaging methods such as mammography, ultrasound, mammography, CT, and MRI have limitations. Mammography, the most widely used method, has a sensitivity ranging from 36% to 98%, with significant false positive and negative results. Furthermore, despite advancements in imaging technology, its application is limited by unnecessary radiation exposure and biopsies (7, 8). Invasive tissue biopsy, the current gold standard for breast cancer diagnosis, fails to capture sufficient information about the tumor’s overall characteristics and is not suitable for dynamic monitoring of cancer development and treatment efficacy (9, 10). Consequently, new diagnostic technologies are urgently needed to overcome these limitations. The latest advancements in less invasive liquid biopsy, driven by new technologies, are leading the way toward precision medicine. Components of liquid biopsy include CTCs, microRNA, extracellular vesicle (EV), proteins, and metabolites secreted by tumor cells (11). As a minimally invasive approach, liquid biopsy is highly valued for its ability to reflect the overall heterogeneity of systemic tumor burden, monitor disease progression, and evaluate treatment efficacy in real-time (12).

SERS is a versatile analytical technique that provides rich molecular information, high sensitivity, narrow spectral bandwidth, strong multi-detection capability, and minimal interference from water (13, 14). Consequently, it holds great promise as a detection tool for disease diagnosis by analyzing body fluids. Notably, SERS has shown practicality in breast cancer diagnosis, as evidenced by recent studies. The mechanisms underlying SERS can be broadly classified into two categories: electromagnetic enhancement (15) and chemical enhancement (16). In summary, the performance of SERS biosensors relies on various components, including the substrate, reporter molecule, stabilizer molecule, conjugated material, and fabrication method. Numerous studies have been conducted on the SERS detection of breast cancer biomarkers, aiming to enhance the sensitivity, specificity, and repeatability of SERS. These improvements have been achieved through strategies such as enhancing substrate affinity, employing labels for detection, and targeting substrates with oligonucleotides.

## 2 SERS detection of tumor biomarkers

### 2.1 Exosomes

Exosomes (EXO), ranging in size from 30-150nm, are extracellular vesicles that have been extensively studied for their involvement in various aspects of cancer (17, 18). including initiation, metastasis, pre-metastatic niche preparation, angiogenesis, immunity, and drug resistance (19). These vesicles carry specific biological and genetic information from their parent cells, selectively enriching certain nucleic acids, proteins, and lipids, thereby providing a comprehensive compositional profile related to cancer pathogenesis and status (20). Notably, characteristic miRNAs found in the nucleic acid cargo of exosomes have been associated with breast cancer invasiveness (21), angiogenesis (22), metastasis (23), and drug resistance (24). Moreover, the expression of exosomal proteins, such as PD-L1, has been closely linked to cancer occurrence, development, and prognosis (25, 26). The stability of exosomes, attributed to their lipid bilayer structure, has attracted attention as a potential biomarker (26–28). However, the heterogeneity of exosomes poses challenges in isolating them from complex biological samples and conducting accurate analyses. Currently, there are two broad categories of techniques for detecting breast cancer exosomes: targeting molecules on the exosome surface using methods such as protein imprinting, enzyme-linked immunosorbent assay (ELISA), polymerase chain reaction (PCR), and various optical approaches; and comprehensive analysis of exosomes, which involves complex instruments and datasets, making clinical application difficult.

In the subsequent sections, we will delve into the most recent studies that employ Surface-Enhanced Raman Spectroscopy (SERS) in conjunction with other approaches to identify exosomes. This integration presents a hopeful avenue for diagnosing breast cancer in its early stages, albeit with the caveat that further enhancements are necessary.

#### 2.1.1 Studies enhancing SERS sensitivity

##### 2.1.1.1 Modified substrate

Substrates play a crucial role in capturing biomolecules, and their capacity varies depending on the substrate. Noble metals such as gold and silver are commonly used in SERS for the detection of breast cancer exosomes due to their ability to enhance sensitivity through local surface plasmon resonance (LSPR) (29). In addition, various other properties of the substrate, such as the size, shape, number and uniformity of nanoparticles (NP) or metals, strongly influence the function of SERS (30). The exposure of a metal surface to a laser beam scatters conduction electrons into frequency electrons, also known as plasmons, that generate an additional electric field corresponding to the electrons of the laser. In nanostructures, these electrons oscillate all over the surface and, thus, are commonly defined as localized surface plasmons, thereby increasing the intensity of the local electromagnetic field, a phenomenon known as localized SPR(LSPR). Given that LSPR depends on the NP size, smaller NPs display poor polarization and result in loss of LSPR properties. Spherical NPs have lower efficiency because of the uniform spread of electric field density (hotspots). Changing the shape from spherical to cubical NPs improves SERS efficiency, mainly because of the focused non-uniform field close to the sharp ends (31). Importantly, more hotspots are created with NP aggregation compared with single NPs (32). In a study by Ferreira et al. (33), low-cost and environmentally friendly SERS substrates were developed. Specifically, *in-situ* synthesis of silver nanoparticles was performed, and these particles were anchored on bacterial cellulose (BC) membranes. The use of nata de coco as a source for BC film production offers a cost-effective and simple alternative to traditional methods. When combined with Principal Component Analysis (PCA), this approach can effectively distinguish exosomes from the MCF-10A and MDA-MB-231 cell lines, demonstrating improved peak differentiation, reduced background noise, and enhanced detectability of analytes due to minimal interference from cellulose peaks. Furthermore, advancements in nanofiber alignment achieved through nano-structuring techniques have enhanced the reproducibility of SERS signals when compared to ordinary cellulose.

Various methods facilitate interactions between exosomes and nanoparticles, including physical anchoring to solid carriers or chemical reaction mediation in a suspension. However, these simple methods can often result in irregular arrangements, leading to inconsistent SERS vibrational spectra. To address this issue, advanced nanofabrication techniques have been employed to improve physical anchoring. Additionally, advancements in nanomachining technology have allowed for precise customization of SERS substrates. Pramanik et al. (34) have reported a heterogeneous SERS platform that employed plasmonic gold nano-stars attached to two-dimensional graphene oxide, resulting in significant Raman enhancement. This platform successfully generated nano-sized “hotspots” through plasmon-exciton coupling, thereby greatly improving Raman efficiency (**Table 1**).

**Table 1:** Detection Limits (LOD) and Dynamic Ranges of Different Methods for Detecting Breast Cancer Exosomes.

Method	LOD/ml	Dynamic range/ml
Ti3C2Tx Mxene-based (35)	20.74	10^2^ to 10^6^
Dual-Aptamer-Assisted SERS Biosensor (36)	1.5 × 10^2^	10^2^ to 10^8^
GO-GNS-based (31)	4.4 × 10^2^	10^2^ to 10^5^
droplet digital Exo ELISA (37)	10^3^	10 to 10^5^
superhydrophobic porous SERS (38)	10^6^	10^6^ to 10^9^
Gold Nanorods and a Miniaturized Device-based (39)	2 × 10^6^	10^6^ to 10^8^
SERS-Lateral Flow Strip Biosensor (40)	4.8 × 10^6^	10^7^ to 10^11^
iREX biosensor (41)	2.9 × 10^7^	10^8^ to 10^12^

The detection of large biomolecules, such as proteins and nucleic acids, is typically conducted after drying on SERS substrates. However, this transition from the hydrated state to the dry state often leads to significant structural changes, resulting in increased spectral variability (38). Yang (36) and Tian (42) investigated the transition state between wet and dry states during the evaporation process of metal nanoparticle sols. In this state, a three-dimensional liquid “hotspot” matrix is formed, substantially increasing the intensity of SERS signals. Additionally, a super-wetting superhydrophobic porous SERS platform (43) addressed limitations by confining exosomes to ordered nanopores, ensuring uniform distribution for stable and reproducible SERS signals (**Table 1**).

Surface modifications are necessary to enhance the capture efficiency of exosomes by simple SERS substrates. Previous studies have focused on constructing trapping substrate-exosome-detection probe structures through immunoaffinity. However, the use of antibodies presents challenges in terms of preservation, stability, and their impact on detection. Therefore, recent studies have shifted towards the development of aptamers and capture molecules that target the exosomal phospholipid membrane structure non-specifically. Zhang et al. (44) developed a dual-aptamer ratio SERS biosensor that demonstrated high affinity and specificity for surface proteins (Epithelial cell adhesion molecule (EpCAM) (45), Prostate specific antigen (PSA) (46), carcino-embryonic antigen (CEA) (47) etc) on exosomes derived from breast cancer cells, resulting in improved sensitivity (48). The strategy involved linking the aptamers targeting two proteins (EpCAM and EGFR2) with 3’ end-modified Rhodamine through short complementary DNA, forming V-shaped DNA. This V-shaped DNA was attached to the Au@Ag NPs/GO substrate surface to selectively recognize exosomes with these two proteins. This approach provided numerous DNA adsorption sites on the GO layer, enhancing sensitivity. In terms of SERS detection of exosomes, multiple Raman probes or machine learning models are typically employed to achieve more precise diagnoses (49). However, these methods often involve cumbersome procedures and lack adequate sensitivity for early cancer diagnosis. In contrast, proportional SERS can effectively reduce the impact of background fluctuations, resulting in high sensitivity, accurate quantification, good reproducibility, and eliminating the need for nucleic acid amplification (50, 51). Thus, proportional SERS holds significant application value in the early diagnosis of cancer.

Sandwich detection methods, such as those that incorporate aptamers, can result in decreased detection efficiency due to spatial blocking (52). To overcome this limitation, a SERS strategy was developed for the sensitive detection of exosome PD-L1 (53). This approach utilized the non-selective recognition ability of MXene and the specific recognition ability of Au@MPBA@SiO. MXene was applied onto a gold chip, facilitating its interaction with the phospholipid membrane of exosomes. By doing so, the MXene was able to non-selectively capture exosomes, effectively overcoming the issue of spatial blocking (54). This novel method significantly enhanced the detection efficiency and sensitivity. Additionally, Daassi et al. (55) have demonstrated the significance of ExoPD-L1 in tumor immune evasion, further supporting the relevance of this platform’s ability to specifically recognize PD-L1 on exosomes. Notably, distinct Raman spectra were observed between healthy individuals and patients, as well as between different cell lines, validating the potential of this approach for distinguishing PD-L1 levels in exosomes from breast cancer patients compared to those of the normal population and between different cell lines.

##### 2.1.1.2 SERS tags

Raman tags have the ability to bind to specific target molecules, resulting in the generation of strong and easily identifiable Raman signals (35). Numerous SERS tags with diverse structures and chemical properties have been documented in the literature. Some examples include 4-mercaptobenzoate (56), 4-Pyridinethiol (57), 4-MBA (58), and 4-Aminothiophenol (59). The utilization of multiple Raman tags not only facilitates the achievement of narrower Raman bandwidths but also expands the range of targets that can be analyzed. Consequently, this approach enhances the sensitivity and specificity for molecular analysis of exosomes (56–58).

Kwizera et al. employed the use of the organic dye QSY21, specifically designed as a Raman reporter label, to successfully monitor eight target proteins (EpCAM, CD44, HER2, EGFR, IGF1R, CD81, CD63, and CD9) found in exosomes. Their findings exhibited a strong correlation with ELISA, boasting an R^2^ value of 0.97 (60). Additionally, the researchers employed 3D printed array templates for the capture of exosomes via antibody arrays. This innovative approach provided rapid results, with a remarkable turnaround time of just 2 hours, making it highly promising for potential applications in clinical testing (**Table 1**).

**Figure 1:**
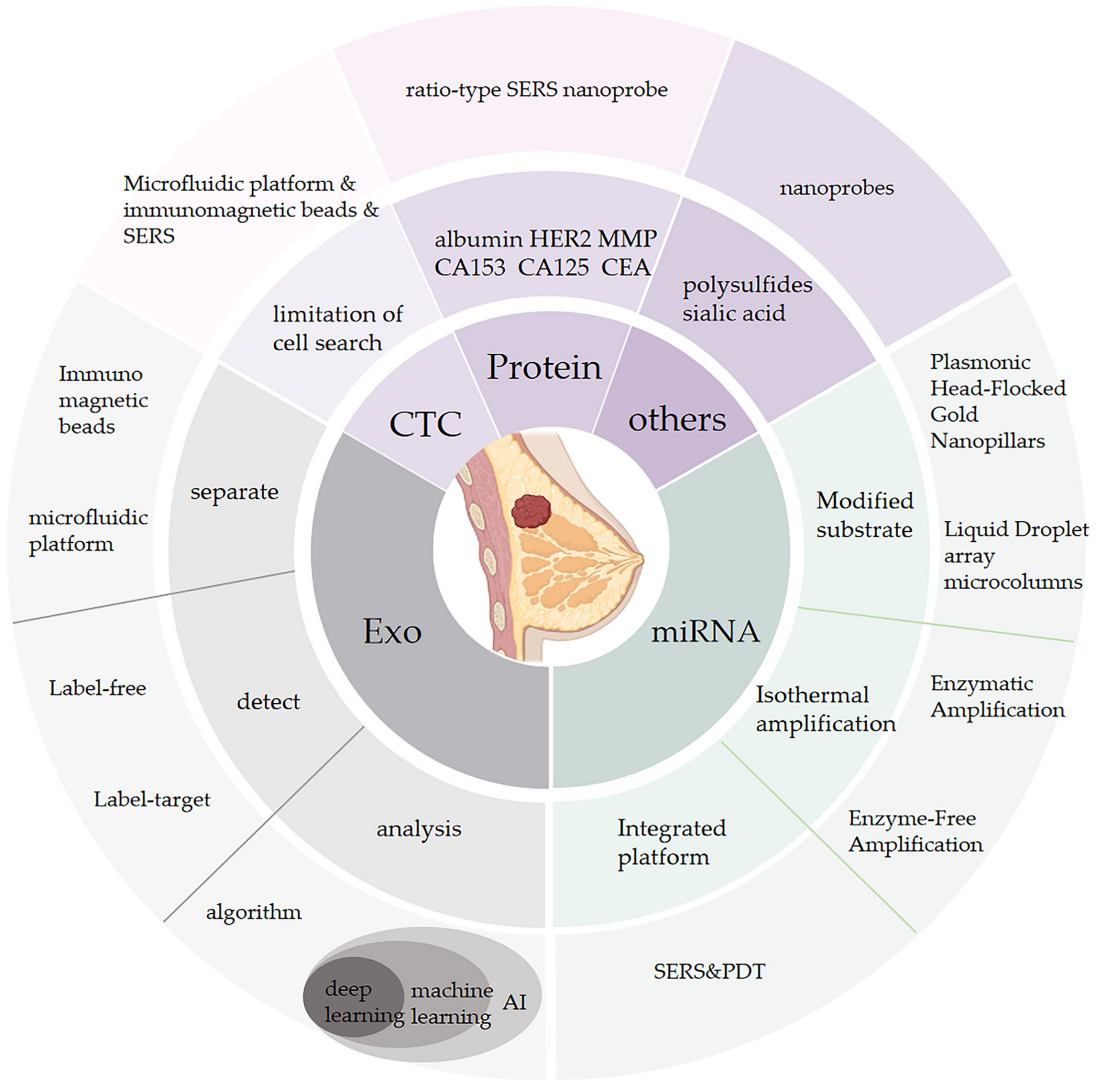
This figure Outlines the novel SERS biosensor technology in terms of breast cancer biomarkers including exosomes, circulating tumor cells, mirnas, various proteins, etc.

On account of the co-expression of proteins of exosomes and subtle variations in expression across different cancer subtypes, Su et al. (61) developed the iREX (Integrated Raman Spectroscopy EXO) biosensor, which is capable of multiple quantitatively analyzing proteins of exosomes (MUC1, HER2 and CEA) in clinical plasma samples from breast cancer patients (**Figure 1**). The study revealed distinct expression profiles of these proteins in different cell subtypes, providing a precise molecular diagnosis of breast cancer subtypes. The iREX biosensor not only ensures accurate detection and diagnosis, but also minimizes false negative results by overcoming the hook effect (39, 41, 62). This biosensor’s ability to simultaneously detect multiple proteins from exosomes on a single vertically flowing NC membrane further enhances its detection capabilities. The study demonstrated that MUC1, HER2, and CEA had distinct expression levels in exosomes derived from MCF-7, SKBR-3, MDA-MB-231, and BT474 cell lines, and these findings were validated using clinical serum samples (**Table 1**).

In summary, the utilization of SERS in conjunction with a diverse range of strategies offers significant improvements in sensitivity, specificity, and reproducibility for the analysis of exosomes derived from breast cancer. These approaches hold great potential for early detection of cancer, thereby facilitating advancements in clinical practice. Nevertheless, it is important to emphasize that further enhancements in SERS technology are imperative to achieve optimal results.

#### 2.1.2 SERS combined with exosome isolation technology

Efficient and reliable methods for the isolation and identification of exosomes have been highly regarded, as competitive adsorption of other molecules to the metal surface can lead to challenging SERS spectra analysis. The integration of exosomes with reporter tags for Raman signals presents challenges for exosome isolation. Inadequate separation may result in the retention of excessive free Raman signal reporter tags, leading to false-positive outcomes (63). Immunomagnetic bead separation technology offers immunoaffinity-mediated exosome recognition and convenient magnetic collection, enabling efficient exosome enrichment. Li et al. (64) combined immunomagnetic bead separation technology with SERS, establishing a magnetic SERS platform. This platform demonstrated sensitive and specific detection of exosomes, enabling differentiation between two distinct breast cancer cell lines (MCF-7 and MDA-MB-231) or exosomes from serum samples with 100% sensitivity and specificity within a 95% confidence interval.

Kwizera et al. (60) constructed a droplet-based immunoassay method for the quantification of exosomes. In this method, the trapping antibody was attached to magnetic beads, enabling the adsorption of target exosomes. Subsequently, the magnetic beads were encapsulated within microdroplets using a microfluidic platform. The concentration of exosomes was estimated by measuring the fluorescence emission resulting from enzyme-catalyzed reactions. This technique exhibited the ability to differentiate the expression levels of target proteins on individual exosomes within the droplets (**Table 1**).

#### 2.1.3 Statistics and artificial intelligence

The complex composition of exosomes leads to intricate vibrational spectra patterns. To achieve more precise analysis of these spectra, SERS in combination with multivariate mathematical and statistical methods such as principal component analysis (PCA), partial least squares discriminant analysis (PLS-DA), and artificial intelligence have been employed. Xie et al. (65) utilized spectral deconvolution of complicated SERS spectra (66) collected from a SERS sensor to quantify serum exosomes. They applied the MCR-ALS algorithm for spectral separation, enabling dual detection using SERS. Furthermore, they accurately determined the concentrations of SKBR and MCF exosomes in clinical serum samples across various pathological conditions, thus demonstrating the clinical applicability of this method.

**Figure 2:**
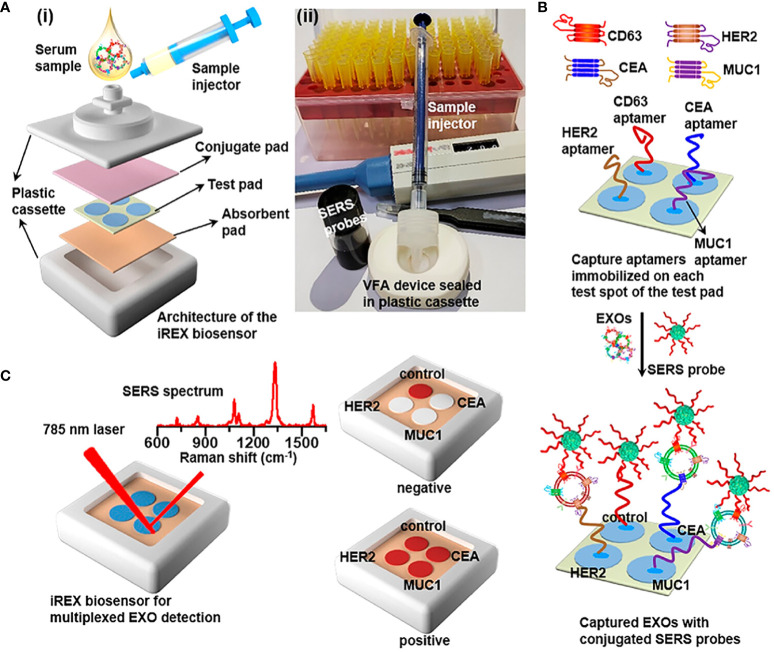
**(A) ** (i) Overview of the architecture and (ii) the real image of the iREX biosensor, which comprises a sample injector, a VFA device and a plastic cassette. The VFA device is stacked with a conjugate pad, a test pad of four spatially separated test spots and an absorbent pad orderly from the top to the bottom. **(B)** Illustration of multiplexed detection of exosomal proteins in serum samples. The aptamers immobilized in the corresponding test spots capture various target EXOs through the specific binding to the corresponding exosomal proteins, and SERS probes are bound onto EXOs via the specific recognition of the CD63 conjugation aptamer to the common exosomal CD63, forming sandwich complexes for the subsequent SERS profiling. **(C)** Schematic of the iREX biosensor based multiplexed detection of exosomal proteins in the presence of no EXOs (negative test) and multiple EXOs (positive test).

**Figure 3:**
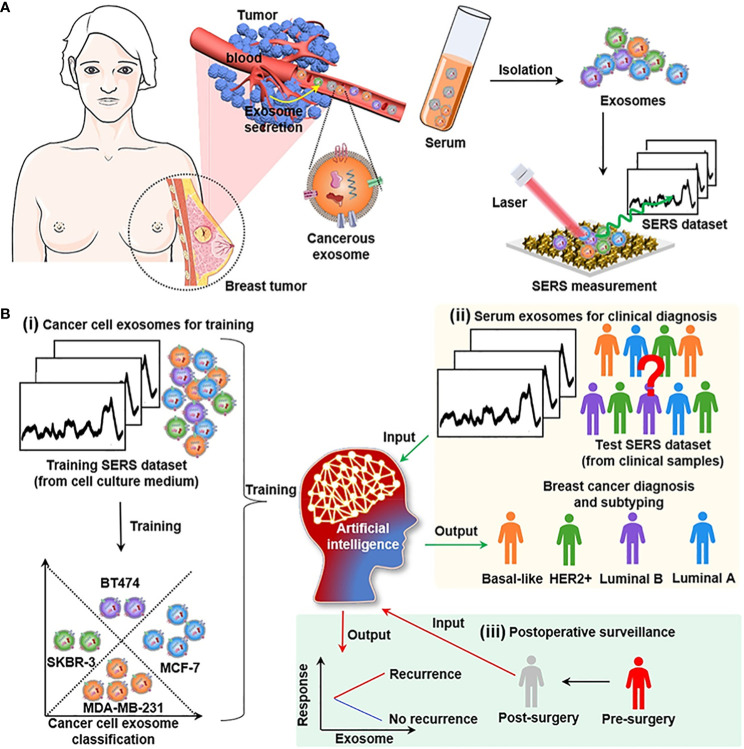
Overview of Deep Learning-Assisted SERS Spectroscopic Analysis of Serum Exosomes for Diagnosis and Postoperative Assessment of Breast Cancer. **(A)** The schematic workflow involving the collection of clinical samples from patients, isolation of serum exosomes, SERS measurement, and deep learning-assisted analysis. **(B)** The deep learning model for cancer diagnosis and postoperative assessment in which the ANN algorithm is first trained and validated using SERS data from cancerous exosomes derived from different cancer cell lines and then employed for analysis of serum exosomes from patients. Adapted from Artificial Intelligent Label-Free SERS Profiling of Serum Exosomes for Breast Cancer Diagnosis and Postoperative Assessment by Y. Xie, X. Su, Y. Wen, **(C)** Zheng, M. Li, Nano Lett. 22 (2022) 7910–7918. https://doi.org/10.1021/acs.nanolett.2c02928.

Additionally, Xie et al. (65) reported an artificial intelligence-assisted SERS strategy for label-free spectral analysis (**Figure 2**). **Figure 3** The artificial neural network (ANN) algorithm was employed to capture the characteristic composition spectrum of serum exosomes in the SERS dataset. This algorithm demonstrated excellent predictive capabilities for distinguishing exosomes originating from different breast cancer cell lines (MDA-MB-231, MCF-7, BT474, and SKBR-3) as well as exosomes from human patients with different cancer subtypes. To assess the efficacy of the strategy, the Mahalanobis distance between the exosome clusters derived from serum samples and those from cancer cells was measured, enabling a quantitative evaluation of the similarity between the plasma and cancer cell exosome data. The results indicated that the relative similarity was reduced following surgical removal of tumors, suggesting the necessity of surgery in the context of breast cancer. Overall, this deep learning-assisted SERS integration strategy demonstrates the potential to accurately diagnose and prognosis breast cancer through the analysis of exosomes (**Table 1**).

In a related study, Shin et al. (37) presented a deep learning approach using a CNN model to detect and classify cancer specimens, as well as determine the specific type of cancer. The method proved successful, achieving accurate differentiation among six different early cancer types, with diagnostic sensitivity and specificity surpassing 90%. While the inclusion of additional training samples and the resolution of the SERS detection chip manufacturing issue are necessary, the system offers a straightforward and rapid approach that requires minimal samples, thus offering a convenient tool for early diagnosis by clinicians.

### 2.2 Circulating tumor cells

CTCs constitute a diverse group capable of generating metastatic tumors in distant sites through epithelial-mesenchymal transition (EMT) (40, 67–69). Numerous studies have established an association between CTCs and adverse prognostic indicators such as poorer prognosis, reduced disease-free survival, and overall survival rates in breast cancer (70–73). SWOG trial (SWOG protocol S0500) results (74) highlighted that modifications in chemotherapy did not yield a significant impact on the overall survival of patients with elevated CTC levels compared to those without. While CTCs have shown promise as an independent prognostic indicator with clinical validity (dividing a population into distinct groups with markedly different clinical outcomes), their clinical utility in enhancing patient outcomes remains elusive.

The CellSearch technology is widely regarded as the gold standard for detecting and separating CTCs in breast cancer. This method targets the epithelial marker protein EpCAM on the surfaces of CTCs. However, CTCs that express EpCAM to a lesser extent or not at all may go undetected, leading to false negatives. To address this limitation, a new microfluidic chip was developed, known as the antibody-functionalized microfluidic (AFM) chip. This chip is capable of capturing not only EpCAM but also CK19, CD45, and DAPI, which enhances the accuracy of CTC detection (75). Another study by Wilson et al. (76) demonstrated the use of a microfluidic platform combined with immunomagnetic separation technology for SERS detection of CTCs in breast cancer. SERS is a highly promising technique for multiple detections due to its narrow peak, addressing the disadvantage of CellSearch, which only targets EpCAM. The use of immunomagnetic separation in a microfluidic device minimizes sample contamination and loss, while its automation reduces human intervention and operational errors. In contrast to quantitative methods for miRNA detection, this platform utilized chemometric least squares (CLS) regression to calculate weight factors for each label. These weight factors correspond to the relative intensity of SERS spectra of nanotags on cells compared to the reference. The authors of this study utilized SERS spectra of precisely defined nanotags to analyze individual cells. By quantifying the SERS signal, they were able to determine the quantity of nanolabels bound to surface proteins, thus enabling the differentiation of various types of breast cancer cells. This platform, which combined microfluidics, immunomagnetic separation, and SERS technology, proved to be an efficient method for detecting CTCs in breast cancer.

### 2.3 miRNA

MicroRNAs (miRNAs), which are small non-coding RNAs, are actively involved in regulating key cellular processes such as cell proliferation, differentiation, apoptosis, and cancer development (77). Interestingly, numerous miRNAs are found within chromosomal regions associated with tumors (78). In particular, miRNAs exhibit significant expression variances between normal and tumor tissues, influencing critical aspects of breast tumor cell proliferation, infiltration, and migration (79). Notably, miR-21 has been reported to be upregulated in human breast cancer cell lines and is crucial throughout all stages of breast cancer pathogenesis (80, 81). Additionally, miR-221/222 clusters (82), along with miR-9, miR-10b, miR-29a, among others, are found to be overexpressed in breast cancer, while miR-30a, miR-31, miR34, miR-93, miR-125, and miR-126 are down-regulated (83–87).

Currently, common methods for detecting miRNA include probe-targeted hybridization or amplification techniques. The former includes northern Western blotting and microarray chip technology, while the latter includes RT-qPCR and sequencing. Northern blotting (88) is considered the gold standard for miRNA detection, but it is time-consuming, has low sensitivity, and requires expensive reagents. RT-qPCR (89) is highly automated, but designing miRNA primers is challenging due to base pairing mismatches during amplification (90). Sequencing technology is suitable for mass screening of early-stage breast cancer, but it is also time-consuming and costly (91, 92). Microarray (93) Target labeling is challenging due to the short probe target (94). Furthermore, miRNA has intrinsic characteristics such as short sequences, highly homology (95), low abundance, (accounting for only 0.01% of total RNA in plasma) (96, 97), and it exhibits a dynamic range of at least four orders of magnitude (98), requiring a highly sensitive detection method. As mentioned above, SERS has been shown to possess advantages such as ultra-high sensitivity, narrow spectral bandwidth, and strong multiple detection capability, making it a promising tool for miRNA detection.

#### 2.3.1 Enhancing SERS sensitivity

In the context of miRNA detection, similar to exosome detection, innovative techniques are utilized in surface-enhanced Raman scattering (SERS) to enhance sensitivity. These methods include the use of novel substrates, targeted labels, and oligonucleotides. Droplet detection (99) presents several advantages, such as compartmentalization, small volume analysis, and high throughput capacities. The integration of SERS with liquid droplet arrays markedly minimizes liquid volume requirements. In contrast to super-wetting microchips described in exosome detection (100, 101) in exosome detection, liquid droplet array microcolumns effectively secure and concentrate small-volume droplets. Homogeneously dispersed miRNA within these arrays can significantly amplify SERS signal intensity (38, 102, 103). This platform (104)has successfully distinguished four breast cancer-related miRNAs, demonstrating promising applications in early breast cancer diagnostics.

Lee et al. (105) demonstrated the development of a SERS biosensor utilizing plasma-headed velvet gold nanorods. This unique configuration induced the aggregation of neighboring nanorods, leading to a reduction in the interparticle gaps and the formation of self-assembled hotspots (106, 107). The biosensor exhibited remarkable sensitivity, enabling the detection of miR-21, miR-222, and miR-200c of exosomes with low limits of detection, as well as a wide dynamic range, without the need for amplification processes.

To achieve the stability of the interaction between the capture probe and miRNA, oligonucleotide-based strategies, specifically hybridization, were employed. The sandwich hybridization strategy (108–110) proved effective only when the target miRNA sequence perfectly complemented both Locked Nucleic Acid (LNA) probes. Due to the separation of the Raman dye from the substrate caused by the hybridization reaction between the DNA probe and the amplified miRNA product, the Raman signal weakened, resulting in a gradual decrease in the height of the Raman signature peak with increasing miRNA concentration. Therefore, miRNA can be quantified through the fitting of a concentration-dependent Raman intensity curve, allowing for a precise quantitative analysis of miRNA.

#### 2.3.2 SERS combined with isothermal amplification strategies

##### 2.3.2.1 Enzymatic amplification

In contrast to exosome detection, several studies have reported strategies that combine isothermal amplification of miRNA with SERS. Xu et al. (111) described a sandwich SERS platform utilizing Au@Ag core-shell nanorods. A significant feature of this sensor is the integration of double-stranded specific nucleases (DSNSA) for signal amplification. DSN is a protein enzyme known for its selective degradation of double-stranded DNA (112, 113). This unique property makes it highly advantageous for miRNA detection. Utilizing the hydrolysis reaction of DSN, the target molecule miRNA can be recycled and reused, allowing for the involvement of trace amounts of miRNA in multiple DSN digestion reactions (114), which improved the sensitivity and amplified the signal. By monitoring Raman signal attenuation, the sensor simultaneously measured miR-21, miR-155, and let 7b through three different Raman reporting tags, respectively.

Meng et al. (115) reported a novel approach to enhance the sensitivity of as SERS biosensor by employing isothermal amplification of microRNA (miRNA) using a nuclease enzyme. In contrast to existing techniques, this method generates a three-dimensional (3D) SERS holographic image through computer processing, which enables visualization, offers excellent reproducibility, and reduces processing time (116). The developed biosensor successfully detects nine breast cancer-related miRNAs in both standard and clinical samples, exhibiting superior accuracy when compared to RT-qPCR.

##### 2.3.2.2 Enzyme-free amplification

Weng et al. (113) described the application of catalytic hairpin assembly (CHA) technology in a SERS biosensor for the highly sensitive detection of miRNA-21 and miRNA-155. In comparison to enzymatic DNA loop reactions, CHA offers several advantages such as low cost and simplicity in reaction conditions, as well as easy storage (117). Through CHA, DNA molecules generated were capable of binding reporter genes to SERS substrates. Consequently, the intensity of the SERS signal was directly proportional to the concentration of DNA products associated with the target miRNAs. This platform achieved remarkably low detection limits of 0.398 fM and 0.215 fM for miRNA-21 and miRNA-155, respectively, by exploiting the synergistic effects of RNA cycling amplification and SERS “hotspots”.

Zhang et al. (118) proposed a novel Fluorescent-Raman Binary Star Ratio Probe (BSR) that utilizes enzyme-free amplification to enhance the sensitivity of surface-enhanced Raman scattering (SERS). The researchers also conducted fluorescence imaging experiments to evaluate the stability of the BSR probes in complex microenvironments and their potential for imaging single living cells. The concentration of miRNA-203 in single cells determined by the SERS signal intensity ratio of Cy3 and Rox was found to be consistent with previous findings. By implementing dual Raman reporter molecules, the platform effectively reduced background interference and exhibited improved stability in complex biological environments. The probe successfully detected miRNA-203 in single cells, thereby offering a promising tool for precise medical imaging.

Similarly, Wang et al. (119) recently reported the development of a SERS biosensor that utilizes a reverse molecular sentinel (iMS) nanoprobe. This nanoprobe incorporates miRNA molecules, causing the displacement of a single DNA placeholder chain and the formation of a stem-ring structure. Subsequently, the nanoprobe binds to the surface of plasma active nanostars (AuNS@Ag), resulting in a significant enhancement of the SERS signal (ON state). Conversely, when the placeholder chain is intact, the increased distance between the nanoprobe and the nanostars leads to a weaker SERS signal (OFF state). To achieve differentiation between miR-21 and miR-34a without the need for target labeling or subsequent washing steps, a suitable occupying chain is designed with a short poly(T) tail at its 3’ end. This occupying chain demonstrates high thermal stability and consistently generates a stable OFF signal.

##### 2.3.2.3 Limitations and strategies

Coupled with isothermal amplification offers a notable advantage in reducing sample consumption and enhancing sensitivity. Nevertheless, it encounters complexities in intricate biomedical analyses, often necessitating time-intensive reactions. Furthermore, in the enzyme-assisted DNA cycle reaction, there is a susceptibility for enzyme adsorption onto the electrode surface, potentially compromising sensitivity. Microfluidic technology emerges as a viable strategy to address such separation challenges. Wang et al. (94)presented a novel approach that integrates SERS with alternating current and microfluidics (120). The enrichment of TMAS (Triggerable Mutually Amplified Signal) probes in a microfluidic reaction chamber leads to significant fluid flow, which is a result of the combined action of electrothermal and alternating current electroosmotic force. This phenomenon promotes the sensitivity of the reaction, as evidenced by a low limit of detection (LOD) of miR-21 at 2.33 fM. In addition, microfluidic chips that employ AC electric flow technology demonstrate efficient mixing capabilities, resulting in improved uniformity and DNA cross rate (121). This, in turn, significantly reduces the time required for detection (122). A comparison between this approach and the traditional microfluidic method (123), reveals shorter measurement times, higher signal uniformity, and enhanced detection sensitivity and reproducibility.

Furthermore, Zhang (124) et al. presented an innovative multimodal spectral approach for early diagnosis of breast cancer using attenuated total reflection Fourier Transform infrared spectroscopy (ATR-FTIR) and SERS data. The authors employed a set of 32 machine learning models to extract complementary information and enhance the accuracy of miRNA detection. The verification accuracy rate achieved was 95.1% while the test accuracy stood at 91.6%.

#### 2.3.3 Other liquid samples

Research on urine samples, as well as serum samples, is currently being conducted. Kim et al. (125) introduced a platform that utilizes silver nanorods grown on SNP, leading to a significant enhancement in sensitivity for the simultaneous detection of two miRNAs (miRNA-21/155). Through the use of nanocolumns grown on SNPs, this platform is able to detect multiple targets at one point, resulting in improved sensitivity with detection limits (LOD) of 451 zmol and 1.65 amol, respectively. The platform also allowed for the quantification of the miRNAs in various samples, enabling the classification of four cancer cell lines (MCF-7, HCC1143, MDA-MB-231, HCC1954).

#### 2.3.4 Comprehensive diagnostic and therapeutic platforms

In their study, Liu et al. (126) investigated the use of copper phthalocyanine (CuPc) molecules as a diagnostic and therapeutic nanoplatform for breast cancer miRNA detection employing surface-enhanced Raman scattering (SERS). The authors demonstrated that CuPc molecules played a dual role in SERS detection capability and photodynamic therapy (PDT). Firstly, the SERS signal enhancement factor (EF) value of CuPc was estimated to be 7.42×10^4^, which surpassed that of other 2D nanomaterials (127). Secondly, PDT (128, 129) combined with CuPc@HG@BN showed promising therapeutic effects. For instance, when the tumor was at an early stage, complete clearance was achieved with a concentration of only 25 μg/mL of CuPc@HG@BN. However, for tumors that had been growing for 9 days, they observed only a slight reduction in miR-21 levels after 3 days of treatment. Even with a drug concentration of 200 μg/mL, the tumor was no longer eliminated, highlighting the importance of this platform in the early diagnosis and treatment of breast cancer in clinical settings. This study not only employed CuPc, but also implemented a ratio strategy and ring amplification of miRNA to improve the accuracy and sensitivity of the platform. The use of ratio strategy enhanced the early diagnosis capability, while the ring amplification improved the sensitivity of the platform, achieving an intracellular miR-21 reaction concentration as low as 0.7 fM. In summary, this study presents a multifunctional platform for the diagnosis and treatment of breast cancer utilizing SERS, contributing to the advancement of SERS applications in breast cancer research.

### 2.4 Proteins

#### 2.4.1 HER2

HER2 is typically distinguished among different subtypes of breast cancer through assessment using a grading system of four levels based on staining intensity. The current gold standard method, immunohistochemistry (IHC), is relatively inexpensive but lacks objectivity in results (130). Fluorescence *in situ* hybridization (FISH) can detect the HER2 gene copy number in each cell nucleus, but it requires expensive reagents and laboratory equipment (131). SERS possesses highly efficient optical properties such as narrow bandwidth Raman peaks. Murali (132), Xie (133), Mo (134), Verdin (135), Téllez-Plancarte (136), et al. have utilized SERS to detect HER2 levels in samples from various sources. Their proportional grading reduces background interference, yielding detection results highly similar to FISH. SERS enables simultaneous, objective, and quantitative detection of multiple receptor proteins rapidly, presenting a promising means for future receptor protein analysis. Similarly, Kapara et al. (137) conducted research on identifying breast cancer subtypes by detecting different levels of ER and PR expression.

#### 2.4.2 Matrix metalloproteinases

Matrix metalloproteinases (MMPs) constitute a large family of proteases (138). Elevated synthesis and activity levels of MMPs in cancer have been shown to result in alterations in extracellular matrix protein hydrolysis (139). The expression of MT1-MMP (140), MMP-2 (141), and MMP-9 (142, 143) is closely associated with cancer invasion and metastasis. Zhong et al. (144) proposed a ratio-type SERS nanoprobe, where the SERS intensity of a substrate peptide labeled with Rh B on the nanoprobe decreased specifically under MMP-2 digestion, while the 2-NT signal served as an internal standard for proportional imaging. Differential expression of MMP-2 in breast cancer of varying malignancy grades enabled the identification of different breast cancer subtypes. Zhu et al. (145) further localized SERS nanoprobe labeling of MT1-MMP in MDA-MB-231 cells, achieving marked and imaging.

Liu et al. (146) developed a SERS biosensing platform revealing real-time changes in MMP-9 secretion during intercellular communication, offering a new strategy for real-time monitoring of MMP-9 secretion during cell communication processes, laying the foundation for future research in breast cancer microenvironments and metastasis.

#### 2.4.3 Serum albumin

Low levels of serum albumin have high application value in determining the prognosis of breast cancer (147). In previous studies, SERS analysis of serum albumin showed great promise in cancer detection and screening, such as colorectal cancer, gastric cancer, nasopharyngeal cancer, and liver cancer (148–150). Lin et al. (151) used hydroxyapatite particles to separate albumin and then obtained its signal through SERS. Through PLS analysis of collected SERS data and using LDA to determine the functional relationship, they achieved an ideal diagnostic sensitivity (100%) and specificity (97.5%).

#### 2.4.4 Other proteins

The relatively low concentration of biomarkers in the peripheral blood of early breast cancer patients limits many current detection methods, while SERS possesses the advantages of trace analysis and multiple detections. Zhen et al. (120) developed a SERS biosensor based on a microfluidic chip that can simultaneously detect three important breast cancer biomarkers: CA153, CA125, and CEA. It holds significant medical significance for early detection of breast cancer and has great application value. Positive margins are a significant cause of breast cancer recurrence.

Breast cancer cells can overexpress CD47 as a means to evade the immune system. Davis et al. (152) collected data on the level of binding of SERS nanoparticles conjugated with CD47-specific antibodies to various breast cancer cell lines as well as margin specimens during surgery. They successfully differentiated clinically different breast cancer cell lines and tumor samples from normal adjacent tissues, although further exploration of their clinical application value is required.

### 2.5 Other biomarkers

#### 2.5.1 Polysulfides

In contrast to ductal carcinoma *in situ* (DCIS), infiltrating breast cancer (IBC) exhibits a significant increase in polysulfides in the stroma due to the decrease in bisulfide, laying the foundation for distinguishing DCIS and IBC by detecting different levels of polysulfides. Kubo et al. (153) used SERS imaging based on gold nanoparticles to monitor and analyze polysulfides and proposed a strategy for automated diagnosis using machine learning. This provides a potential means for monitoring the hyperplasia of connective tissue occurring in cancer-related stromal regions.

#### 2.5.2 Sialic acid

Sialic acid (SA) is a component that can bind to salivary glycolipids and glycoproteins (154). Its abnormal expression is believed to be associated with the occurrence and development of tumors. Obtaining sialic acid is simpler and less invasive than other biomarkers. Liang et al. (155) developed a SERS molecular strategy based on MPBA@AgNP nanoprobes. They also utilized big data analysis to increase the reliability of SERS data results. Peak ratio at 1074 and 1572 cm^-1^ indicates that the expression level of sialic acid on tumor cells is higher than that on normal cells, thus achieving early diagnosis of breast cancer. Similarly, Hernández-Arteaga et al. (156) validated the increased level of sialic acid in the saliva of breast cancer patients compared to benign tumors using a citrate-Ag-NP-based SERS biosensor. These studies contribute significantly to the field of early diagnosis of breast cancer through the use of sialic acid as a biomarker.

## 3 Conclusions and future directions

At present, several issues hinder the clinical application of SERS in breast cancer detection. Firstly, the utilization of SERS technology to simultaneously detect multiple biomarkers in clinical samples (e.g., tissues, serum) is hindered by prolonged detection time and subtle differences in target biomarkers. To address this, combining the complex data obtained by different SERS sensors with artificial intelligence and statistical technology has emerged as a general trend. However, a lack of standard detection procedures persists. Secondly, exosomes, miRNAs, and CTCS exhibit low abundance and necessitate isolation from other small molecules in body fluids, such as cell fragments, lipoproteins, and protein aggregates. Thirdly, while the amalgamation of SERS with immunomagnetic separation and microfluidic technology partially resolves this limitation, studies on this topic remain scarce. There is insufficient research data to definitively establish which substrate type can generate more hotspots and achieve higher detection sensitivity. Furthermore, although some studies have investigated the use of SERS for detecting breast cancer samples in tears, saliva, urine, and other liquid sources, limited research exists on the detection of exosomes, miRNAs, or CTCS in these samples. Consequently, it is imperative to develop SERS strategies that are capable of detecting exosomes, miRNAs, and CTCS, while also ensuring the acquisition of high-quality and reproducible Raman signals. Consequently, this novel avenue of research focusing on liquid biopsy via SERS holds significant promise for obtaining comprehensive diagnostic information in the future.

## Author contributions

XL: Investigation, Writing – original draft, Writing – review & editing. YJ: Investigation, Writing – original draft, Writing – review & editing. CZ: Conceptualization, Funding acquisition, Supervision, Writing – review & editing.
